# The Atlanta Society of Medicine

**Published:** 1895-01

**Authors:** 


### THE ATLANTA SOCIETY OF MEDICINE.

We are glad to note that the Atlanta Society of Medicine has awakened from its lethargy and shows some evidences of progressiveness which have been lacking for a number of years and which has retarded the usefulness and growth of an institution worthy of the unqualified support and co-operation of every regular practitioner of good standing in this city.

The Society has recently removed from the Fitten Building to spacious quarters in the Young Men's Christian Association Building. This hall has a comfortable and abundant seating capacity, and has in addition, a large and convenient room adjoining, which will be used for the accommodation of the medical library.

This latter project should receive our heartiest encouragement.

A library committee was appointed at a recent meeting and we feel assured no effort will be spared to make this feature of the Society a great success.

The following gentlemen were elected to the various offices for the ensuing year:

Dr. C. D. Hurt, President; Dr. R. R. Kime, Vice-President; Dr. W. L. Champion, Secretary; Dr. L. B. Grandy, Treasurer; and Dr. N. O. Harris, Corresponding Secretary.

